# Radio-luno-triquetral bone-ligament transfer as an additional stabilizer in scapholunate-instability

**DOI:** 10.1007/s00402-020-03690-2

**Published:** 2020-11-29

**Authors:** Luzian C. P. Haug, Tom Adler, Dietmar Bignion, Esther Voegelin

**Affiliations:** Department of Hand Surgery, University Hospital of Bern, 3010 Bern, Switzerland

**Keywords:** Scapho-lunate instability, SL-reconstruction, Bone-ligament transfer

## Abstract

**Introduction:**

Reconstruction of the scapho-lunate (SL) ligament is still challenging. Many different techniques, such as capsulodesis, tendon graft and bone-ligament-bone graft have been described to stabilize reducible SL dissociation. If primary ligament repair alone is not possible, an additional stabilizer is needed to achieve scapho-lunate stability. A new local bone-ligament transfer using half of the radio-luno-triquetral ligament is performed. The direction of traction of the transposed ligament is very similar to the original ligament. Ideal tension can be attained by fixation of the bone block at the dorsal ridge of the scaphoid. The biomechanical stability of this bone-ligament transfer shall be examined biomechanically.

**Material and methods:**

Computed tomography imaging was performed using eight cadaveric forearms with a defined position of the wrist. Axial load was accomplished with tension springs attached to the extensor and flexor tendons. Three series ([a] native, [b] divided SL ligament and [c]) after reconstruction with bone-ligament transfer] were reconstructed three-dimensionally to determine the angles between radius, scaphoid and lunate. The radial distal part including a bone fragment of the radio-luno-triquetral ligament was transferred from its insertion at the distal edge of the radius to be attached to the dorsal ridge of the scaphoid.

**Results:**

SL gap was widened after its transection. Average SL distance was 6.6 ± 1.6 mm. After ligament reconstruction, the gap could be narrowed significantly to 4.2 mm (± 0.7 mm). The movement of the scaphoid and lunate showed significant changes, especially in wrist flexion, fist closure and radial deviation. These deviations could be corrected by the bone ligament transfer.

**Conclusion:**

Reconstruction of a transected SL ligament with a bone-ligament transfer from the radio-luno-triquetral ligament reduces SL dissociation under axial load. The described surgical technique causes low donor-side morbidity and can be considered in addition to improve stability if SL ligament suture alone does not appear sufficient.

**Level of evidence:**

Level II, therapeutic investigating experimental study.

## Introduction

The scapho-lunate (SL) ligament is part of the intrinsic ligaments of the wrist and forms a crescent-shaped interosseous connection between the proximal pole of the scaphoid bone and the lunate. According to Tottermann [1], it is the most important stabilizer of the entire hand. Scapho-lunate ligament instability is one of the dissociative disorders of the proximal carpal row. It is caused by a partial or complete rupture of the SL ligament and represents the most frequent and clinically significant carpal instability. However, only by concomitant injuries of the secondary stabilizers, SL advanced collapse (SLAC) may occur [2]. The surgical treatment aims to restore the carpal alignment and kinematics of the wrist, with the ultimate goal of pain relief and avoidance of degenerative osteoarthritis. Allthough Lavernia et al. and Blatt [3, 4] noted marked improvement after dorsal capsulodesis, a subsequent study by Wyrick noted different outcomes with persisting wrist pain and SL and capitolunate angles is not markedly improved [5]. Moran et al. [6] analyzed the outcome of different SL repair techiques, such as Berger-type dorsal capsulodesis [7] and Brunelli tenodesis [8]. Many patients still experienced moderate to severe wrist pain and radiographic improvements in carpal alignment tended to deteriorate over time. Partial arthrodesis, on the other hand, changes the kinematics and may lead to secondary osteoarthritis over time [9–12]. Direct ligament repair is mainly reserved for the acute and subacute SL injuries. However, quality of ligament stumps is often unsatisfactory, so additional stabilization of the SL interval is required. Instead of a complex ligament reconstruction, a local bone-ligament transfer with low donor-side morbidity is desirable. In this study, we present a new, distally based local bone-ligament transfer using the distal part of the radio-luno-triquetral ligament. The direction of traction of the transferred bony augmented ligament is similar to the original ligament. The tension can be attained by fixation of the bone block at the dorsal ridge of the scaphoid. The aim of this study was to examine the biomechanical behavior of the carpal bones after reconstruction of a dissociated scapholunate interval with this bone-ligament transfer.

## Materials and methods

Eight fresh frozen cadaveric upper extremities (4 female, 4 male; age range 63–78 years) have been prepared as described below after thawing them at room temperature. Donors had voluntarily donated their bodies for scientific purposes after their demise.

**Fig. 1:**
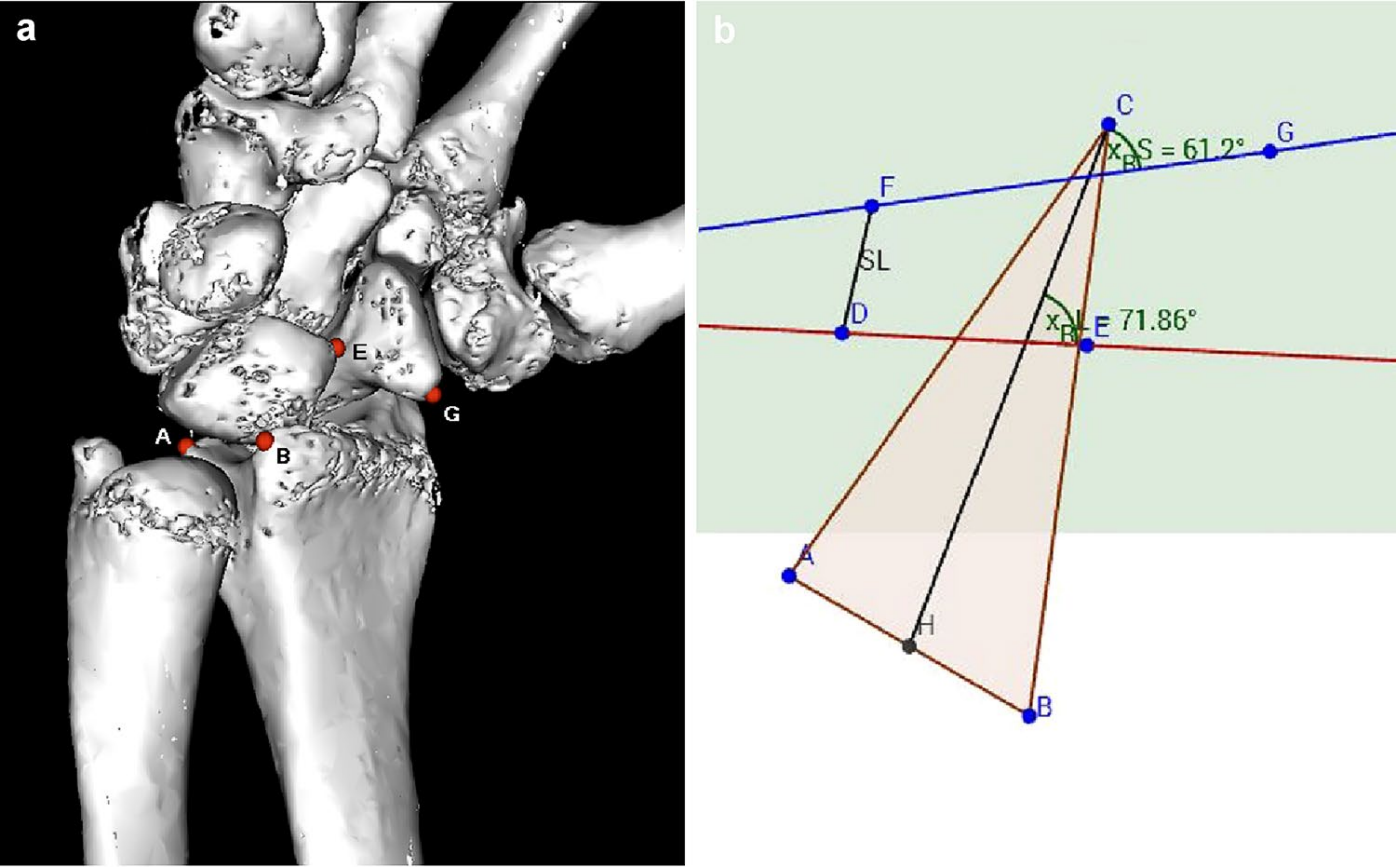
**a** 3D reconstruction with the set markers, visible only on the ulnar side of the radius [A,B], on the palmar side of lunate [E], and shaphoid [G]. **b** Transfer of the markers to the coordinate system: axial view; triangle A-B-C represents distal radius surface. D-E dorsal and palmar lunate horns. F-G represents proximal and distal scaphoid pole. F-D represents SL-Distance

According to a similar experimental setup from Pollock, we decided to use the same weights to simulate wrist movement [13]. Tension springs were attached to the tendons with a predefined tension of 50 N (N). For wrist flexion, flexor carpi ulnaris (FCU) and flexor carpi radialis (FCR) tendons were put under tension, for radial deviation FCR and extensor carpi radialis brevis (ECRB) and longus (ECRL), for ulnar deviation extensor carpi ulnaris (ECU) and for wrist extension ECRB + ECRL and ECU. To simulate a clenched fist, ECU, ECR and tendons of flexor digitorum profundus (FDP) and flexor pollicis longus (FPL) were loaded with 50 N each. The goniometer was used, to ensure that the wrist position was always identical in each series. Computed tomography was performed for each wrist position. The protocol was then repeated after the SL dissection and after reconstruction using the bone-ligament transfer, respectively. After completion of testing in all 8 specimens, CT images of the three series ([a] native, [b] divided SL ligament, and [c]) after reconstruction with bone-ligament transfer] were then reconstructed into 3D models using Osirix software (Osirix Dicom Viewer 8.0, Pixmeo SARL Switzerland). In the 3D model, virtual markers at the radius, scaphoid, and lunate were then set at exactly the same points. To obtain a relative fixed point, three markers were placed on the distal radius: A; dorsal edge of the sigmoid notch, B; palmar edge of the sigmoid notch, C; at the tip of the radius styloid. For the lunate: D; dorsal horn of the lunate, E; palmar horn of the lunate. For the scaphoid: F; dorsal tubercle of the scaphoid, G; tip of the distal scaphoid pole (see Fig. 1). The markers were then transferred to a coordinate system and the angles in three planes could be calculated. The x, y, and z coordinates of the virtual markers were calculated using trigonometric methods (analytic geometry) to measure the angles. Graphical method is realized by using the software GeoGebra [14]. The relative movements of scaphoid and lunate could be determined in the sagittal, coronary, and axial direction. Furthermore, the distance of the scapholunate gap was measured at defined positions of the wrist in flexion, extension, ulnar and radial deviation, and fist closure.

### Statistical methods

Mean values and standard deviations were calculated. Differences between the three series were calculated by use of paired student’s t-test to evaluate statistical significance. Significance was reported as *p* < 0.05. Power analysis of the SL interval difference before and after dissection with a type-I-error value of 0.05 and type-II-error value of 0.1 indicated that a sample size of 8 allows a power of 90%, assuming a standard deviation of the SL interval size of 1 mm [13, 15]. Data were analyzed with SPSS 11.5 for Windows (SPSS, Chicago, IL).

### Surgical technique

**Fig. 2:**
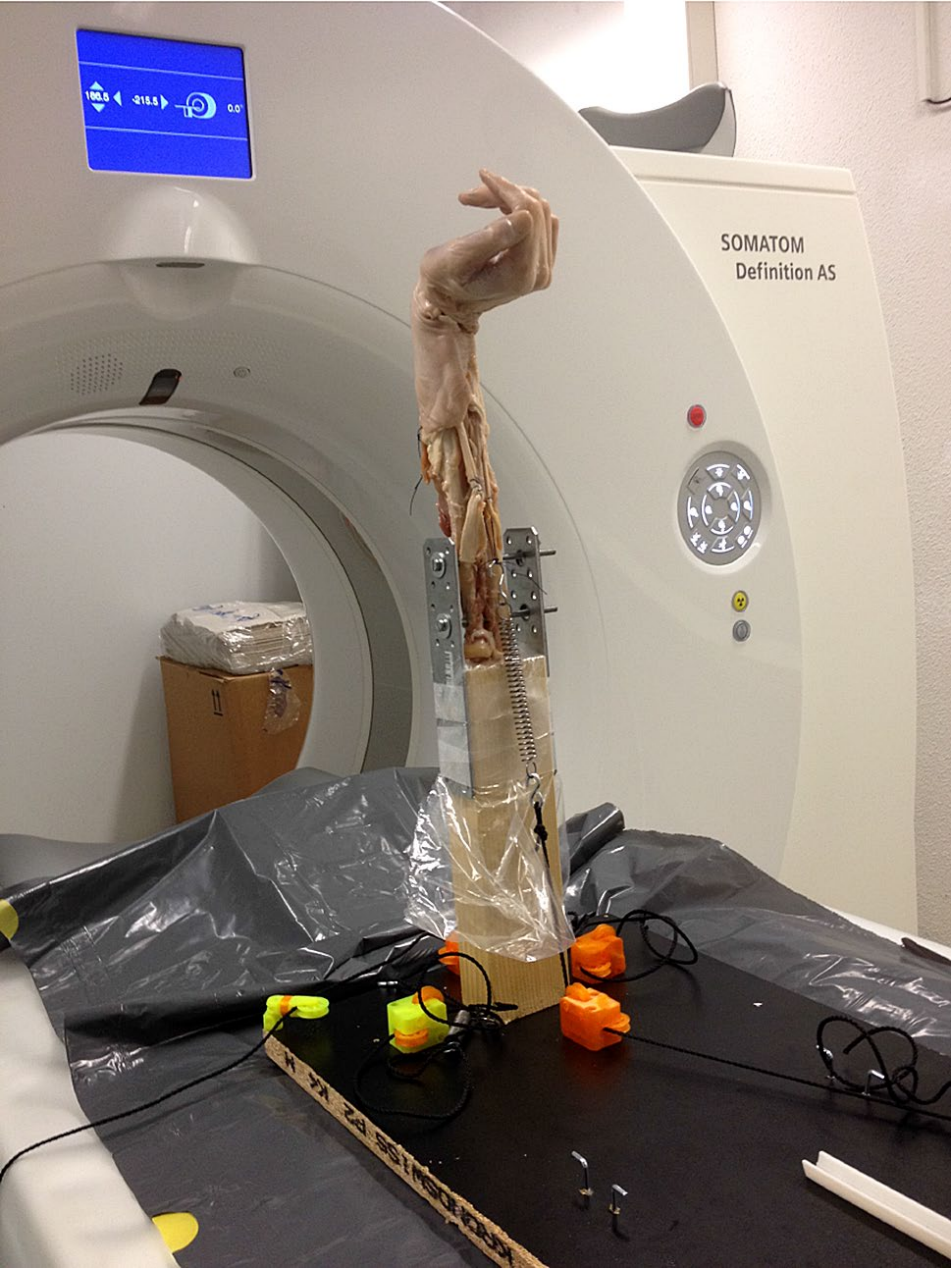
Mounting of the cadaver arm with loaded tendons with tension springs (50 N)

Primarily, the flexor and extensor muscles were removed. The tendons of wrist extensors (ECU and ECRB + L) are attached with a Vicryl thread 1–0 by means of Krackow suture [16]. Similarly, the tendon suture is performed for the FCR tendon and the FCU tendon. In order to simulate the closure of the fist, the FDP and the FPL tendon are sewn side-to-side with a running suture. An osteotomy of the olecranon process of the ulna was performed at the level of the radial head. The forearm was then fixed into the mount with 3.5 mm screws (see Fig. 2).

For the second series [b], the extensor retinaculum was incised over the third and fourth dorsal wrist compartment through a dorsal approach and a radiocarpal arthrotomy was performed by splitting the radiolunotriquetral ligament (RLT) in two halves in direction of its fibers. This exposed the SL ligament, which was then completely transected sharply with a scalpel followed by division of the volar radioscaphocapitate ligament and scapho-trapezial ligament to create SL dissociation [17, 18]. According to previous studies [13, 18, 19], 100 cycles of maximal wrist extension and flexion were performed manually after transecting the SL ligament and its secondary stabilizers to adapt the tissue to the SL instability which had just been created.

**Fig. 3:**
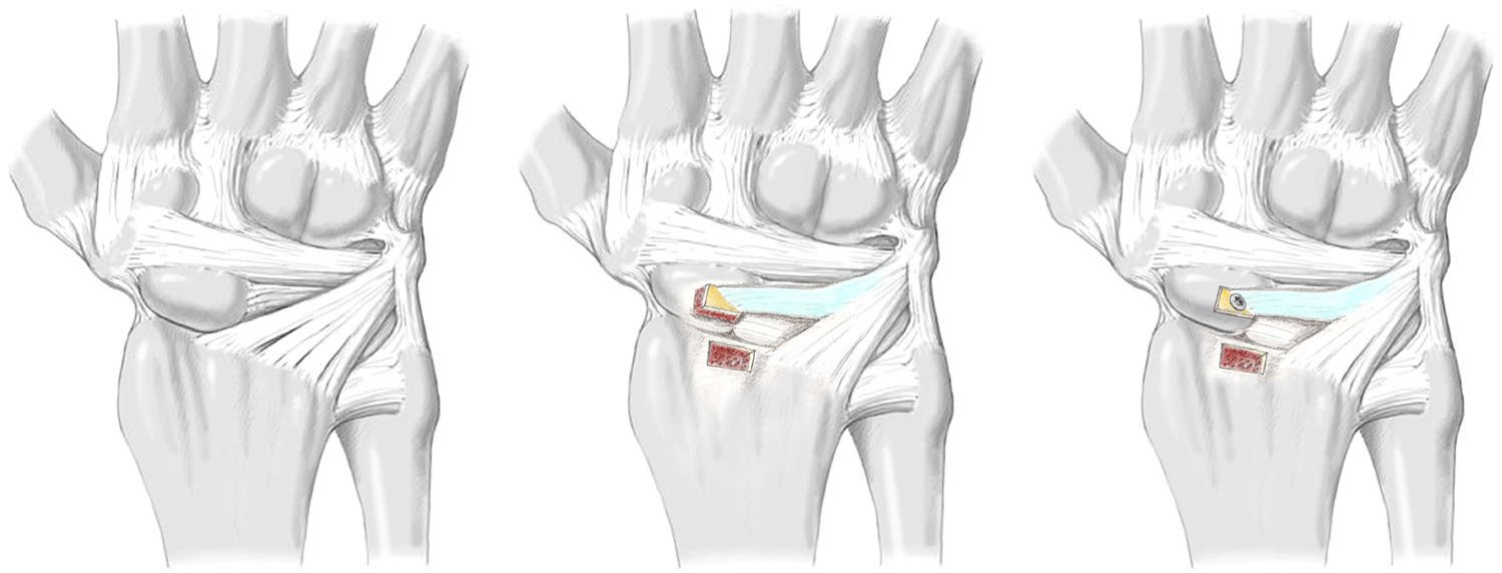
Schematic illustration of the bone-ligament transfer of the radio-luno-triquetral ligament (modified after Lutz et al. [20])

For the third series [c], the previously split radio-luno-triquetral ligament was detached proximally at the margin of the distal radius with a connecting bone block 3 × 5 mm in size using a chisel. A corresponding bone window was removed from the dorsal ridge of the scaphoid and the bone-ligament transfer was fitted (see Fig. 3). In order to withstand the biomechanical tests, the bone-ligament transfer was attached using an intraosseous 0.5 mm wire cerclage. Under real conditions, the bone-ligament transfer would be fixed by screw osteosynthesis. If the bone block is not suitable for a screw, in a clinical setting, an anchor with fibre wire, which can be inserted in the bone window of the scaphoid, can be used to get enough tension to the bone-ligament transfer.

## Results

### Sagittal movement

Sagittal movement means flexion and extension of the carpal bones. In the lateral view, the scaphoid shows significant flexion after separation of the SL ligament, but only in wrist flexion (10.0° (SD 7.1, *p* < 0.05)), radial deviation (18.2° (SD 7.1, *p* < 0.05)), and ulnar deviation (20.5° (SD 7.7, *p* < 0.05). The lunate extends after destabilization of the scapho-lunate interval significantly at clenched fist position (5.8° (SD 4.7, *p* < 0.05)) and wrist flexion (8.2° (SD 6.6, *p* < 0.05)).

After reconstruction of the SL-ligament using bone-ligament transfer, the scaphoid can be significantly reduced to its original position in wrist flexion and radial deviation. The reconstruction had no significant influence of the sagittal movement in the other wrist positions (see Table 1).

**Table 1:** Delta angles between two series (nat = native condition, cut = SL-ligament dissected, OP = reconstruction with bone-ligament-transfer), mean values ± standard deviation

	Δ nat-cut	*p*-value	Δ cut-op	*p*-value
Scaphoid motion				
Sagittal				
Extension	6.8 ± 20.1	0.307	2.8 ± 15.5	0.170
Fist	19.8 ± 13.8	0.134	20.3 ± 7.0	0.073
Flexion	10.0 ± 7.1	0.005	11.0 ± 10.5	0.000
Radial dev	18.2 ± 7.1	0.039	19.7 ± 7.1	0.003
Ulnar dev	20.5 ± 7.7	0.002	10.5 ± 12.6	0.067
Coronal				
Extension	7.7 ± 10.0	0.053	1.4 ± 7.6	0.435
Fist	0.3 ± 8.5	0.312	8.0 ± 6.7	0.158
Flexion	8.3 ± 7.9	0.020	11.9 ± 3.9	0.000
Radial dev	37.4 ± 12.1	0.002	39 ± 26.5	0.001
Ulnar dev	9.9 ± 20.9	0.124	2.0 ± 14.9	0.404
Axial				
Extension	1.1 ± 10.7	0.341	5.1 ± 6.1	0.041
Fist	11.6 ± 15.6	0.207	14.5 ± 6.8	0.091
Flexion	0.2 ± 4.5	0.314	3.9 ± 7.6	0.034
Radial dev	15.9 ± 4.4	0.011	14.1 ± 8.8	0.000
Ulnar dev	2.7 ± 10.3	0.153	17.3 ± 6.8	0.000
Lunate motion				
Sagittal				
Extension	8.8 ± 27.9	0.181	7.0 ± 24.2	0.303
Fist	5.8 ± 4.7	0.006	4.2 ± 6.4	0.053
Flexion	8.2 ± 6.6	0.031	10.1 ± 6.2	0.000
Radial dev	2.7 ± 12.3	0.242	6.4 ± 8.9	0.028
Ulnar dev	6.5 ± 13.1	0.127	10.5 ± 12.6	0.177
Coronal				
Extension	2.9 ± 7.7	0.163	3.4 ± 9.9	0.182
Fist	3.6 ± 9.9	0.166	7.6 ± 8.4	0.019
Flexion	0.2 ± 9.3	0.481	42.9 ± 37.5	0.004
Radial dev	4.7 ± 38.5	0.427	15.0 ± 19.9	0.054
Ulnar dev	12.8 ± 14.9	0.084	5.1 ± 14.5	0.180
Axial				
Extension	2.4 ± 8.0	0.480	3.8 ± 3.6	0.093
Fist	8.2 ± 12.6	0.165	11.2 ± 8.1	0.086
Flexion	4.2 ± 3.5	0.025	0.5 ± 8.7	0.222
Radial dev	7.7 ± 10.1	0.013	3.4 ± 7.1	0.017
Ulnar dev	5.5 ± 6.5	0.050	8.4 ± 7.2	0.004

### Coronal movement

Coronal movement means radial and ulnar deviation of the carpal bones. After separation of the SL ligament, the scaphoid experiences radial deviation during wrist flexion (8.3° (SD 7.9, *p* < 0.05)) and radial deviation (37.4° (SD 12.1, *p* < 0.05)). However, no difference can be seen during other wrist positions. The lunate does not rotate in the coronal plane after SL dissection.

After reconstruction, rotation of the scaphoid is restored both, in flexion (*p* < 0.001) and radial deviation (*p* = 0.002).

## Axial movement

Axial movement means pronation and supination of the carpal bones. Scaphoid bone experiences pronation after cutting the SL-Ligament. This rotation can be seen best in radial deviation (15.9° (SD 4.4, *p* < 0.05)) but less obvious in other wrist positions. Whereas the lunate performs a slight supination movement after the SL ligament has been divided. This rotation can only be detected in flexion (4.2° (SD 3.5, *p* < 0.05)) and ulnar deviation (5.5° (SD 6.5, *p* = 0.05)).

These slight rotations in pronation and supination are corrected after ligament reconstruction, especially in radial (*p* < 0.05) and ulnar deviation (*p* < 0.05).

### Scapholunate interval

**Fig. 4:**
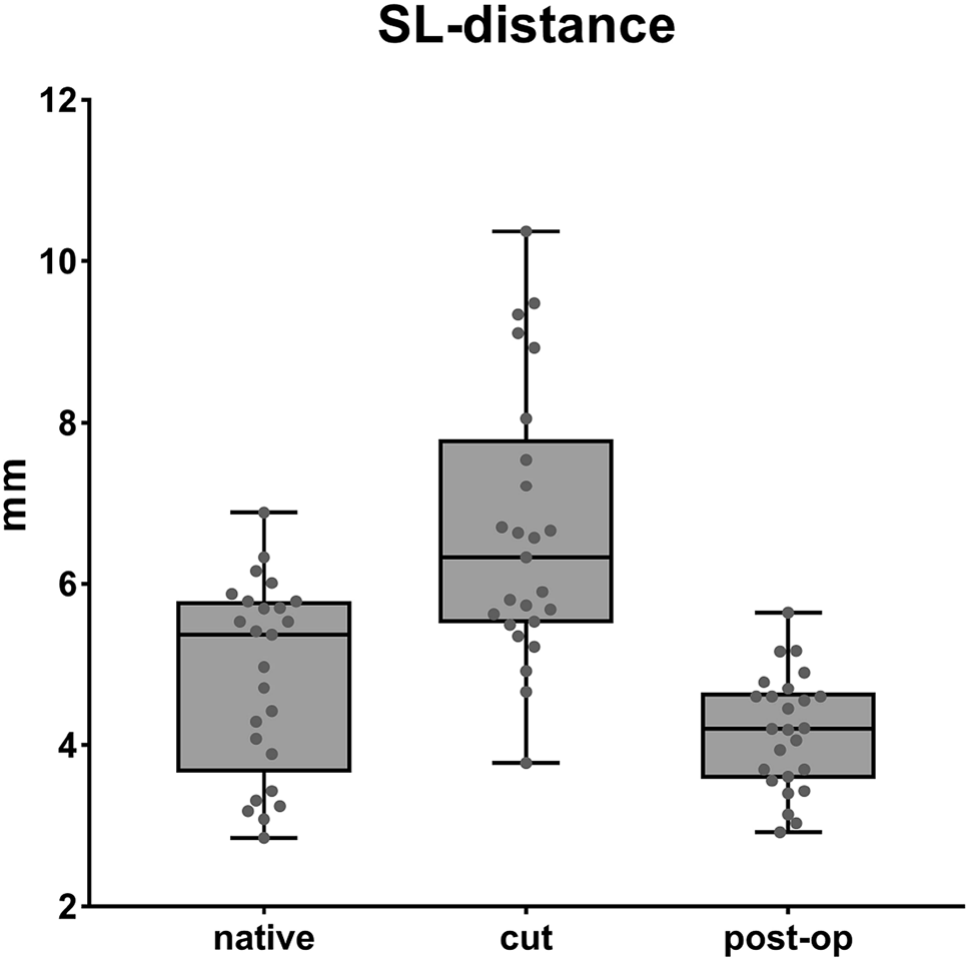
Mean difference of SL distance between the 3 series [a]–[c] in all wrist positions

The mean value of the SL distance, measured between the two markers, seen across all wrist positions, showed 4.8 mm (SD 1.2) in the native group [a]. After dissection of the SL ligament and its secondary stabilizers [b], a SL distance of 6.6 mm (SD 1.6) was measured. This difference is statistically significant (p < 0.001). After reconstruction with the bone-ligament transfer of the RLT ligament [c], the distance was reduced to 4.2 (SD 0.7 mm) (p < 0.001). The mean values in the native group and reconstruction group correlate exactly and show the same range (see Fig. 4). Remarkable is that the SL distance in group [b] is greatest during fist closure, flexion, and radial deviation, but is less prominent in extension and ulnar deviation (7.73 mm (SD 2.2) vs. 5.98 mm (SD 1.4)) (compare Table 2).

**Table 2:** Mean values ± standard deviation

	SL-distance				
	native [a]	cut [b]	op [c]	p [a] vs. [b]	p [b] vs. [c]
Extension	5.30 ± 1.2	6.39 ± 2.4	4.26 ± 0.7	0.091	0.006
Fist	4.25 ± 1.5	6.94 ± 0.8	4.25 ± 0.6	0.060	0.000
Flexion	4.78 ± 1.6	6.88 ± 1.5	3.75 ± 1.1	0.006	0.000
Radial dev	5.15 ± 1.1	7.73 ± 2.2	4.23 ± 0.3	0.003	0.001
Ulnar dev	4.55 ± 0.7	5.98 ± 1.4	4.35 ± 0.9	0.005	0.008

## Discussion

Many surgical procedures have been described to reconstruct SL dissociation. Garcia-Elias et al. advice, how to stage SL dissociation and how to treat it adequately [21]. At stage 2 (complete SL ligament injury with a repairable dorsal SL ligament), direct ligament repair and additional capsulodesis is recommended, if surgery is performed in early stages. At stage 3 (complete non-repairable SL ligament injury with a normally aligned scaphoid), ligament reconstruction should be performed, if no carpal malalignment is present. A bone-ligament-bone reconstruction could be one of the possible procedures [21–23]. Deciding whether a ligament is repairable or not is not always easy. Very often the quality of the ligament is critical for primary repair in non-recent injuries, but there is a pronounced donor-side morbidity with a more extensive reconstruction. Therefore, an additional stabilizer of the sutured SL ligament is desirable.

Many biomechanical studies have investigated different methods of SL ligament repair [13, 19, 24, 25]. The measurements were always carried out using fluoroscopy. The angles between scaphoid and lunate were then determined using a two-dimensional X-ray image. Static radiography alone may miss a dynamic SL dissociation [26, 27]. Intra- and interobserver reliability to recognize carpal instability is discouraging [28]. The angles cannot be measured exactly and measurements are only approximative. Furthermore, in the lateral view, only the sagittal movement of the carpal bones is considered. As we all know, carpal bones follow a complex three-dimensional kinetic, and a two-dimensional image is insufficient to describe this intricate motion. Therefore, a more accurate method should be used for the representation of the movement of the carpal bones. Thus, we analyzed the movements using the 3D reconstructed computed tomography. Moreover, only a momentary situation is taken with the standard x-rays. The displacements at different wrist positions can hardly be detected due to the superimposition of the carpal bones with normal x-rays. That was the reason why we carried out the analysis of the carpal movement using 3D models, to obtain more precise measurements of the complex, three-dimensional movement.

Our results show that after dissecting the SL ligament, the scaphoid follows its natural movement into flexion, especially during wrist flexion, radial, and ulnar deviation. Simultaneously, the lunate follows a dorsal extension movement, again during flexion of the wrist, but particularly during fist closure. It's worth mentioning that these are relative motions to each other. We could demonstrate that the ligament reconstruction neutralizes this abnormal movement after a divided SL interval. During wrist flexion and radial deviation, the scaphoid itself undergoes radial inclination, while the lunate does not perform any particular movement in the coronary plane. This pathological deviation could also be corrected by bone-ligament reconstruction described above. When looking at the scaphoid and the lunate from the axial view, one will be able to recognize a pronation of the scaphoid and a slight supination of the lunate after an SL dissociation has been created. Again, this effect is not observed in all wrist positions.

On the other hand, we could show that the separation of the SL ligament and its secondary stabilizers result in a significant increase of the SL distance in all wrist positions. Interestingly, the SL distance increases more during fist closure, flexion, and radial deviation. With the radio-luno-triquetral reconstruction, the SL distance could be restored in its original position.

Comparing other techniques like Blatt capsulodesis [4], which create a tether form, the dorsal rim of the radius to the distal scaphoid in order to limit scaphoid flexion, our described bone-ligament transfer lead to a more anatomic reconstruction, since the dorsal SL-ligament is strengthened along its original anatomy. It can be assumed that the bone block heals well in the scaphoid under optimal conditions, thus creating a very stable bone-ligament interface.

The modified Brunelli technique as described by Talwalkar, Van den Abbeele, and Garcia-Elias [29–31], tries to correct the flexion of the scaphoid by routing the FCR tendon through te distal volar surface of the scaphoid and securing it to the dorsal pole of the lunate as well as looping it around the radiolunotriquetral ligament. This method addresses both distal and volar secondary stabilizing ligaments, as well as the SL-ligament, which is recommended in higher stage SL instability. Our described technique does not restore secondary stabilizers and is therefore not suitable for chronic and static SL instability. On the other hand, donor-side morbidity by the rather big approach described by the modified Brunelli technique is significantly more serious than our technique.

Other authors suggest a bone-ligament-bone reconstruction to restore SL instability [22, 23]. Donor-side morbidity is again much greater than in our described technique. In addition, the advantage over a free bone-ligament bone graft is that the bone block is still attached on one side with a better blood supply as well. The described technique may also be performed with a mini-open approach together with arthroscopic-assisted SL transfixation.

Limitations of our study include those inherent in cadaver-based studies. Cadaver specimens do not allow tissue healing to assess reconstruction durability accurately. The age of the deceased and their degenerative changes on the wrist may have an influence on the results, particularly since this injury must be treated surgically, principally in younger patients. Although we could show that the SL distance can be reconstructed very well with this technique, the evidence for anatomical carpal alignment is more difficult to realize. For certain wrist positions, this seems to be possible, but for others not at all. This is probably due to the fact that the bone ligament graft does not address the secondary stabilizers, which is not the aim of this technique.

In conclusion, this study showed a more detailed analysis of the pathological movement that occurs in the carpal structure after SL dissociation. Especially during wrist flexion, clenched fist, and radial deviation, an increased aberration of scaphoid and lunate from the native position is apparent. It can be assumed that the stress of the SL interval is greatest in these positions. We also demonstrated that the described radio-luno-triquetral bone-ligament transfer restores the pathological deviations in the corresponding wrist positions. In particular, the widened SL gap could be significantly reduced by the bone-ligament reconstruction.

Since the described surgical technique causes low donor-side morbidity, this surgical procedure can be considered in addition to the primary suture, if the quality of the SL ligament is doubtful. So far, we have little experience in the clinical use of this new bone-ligament transfer. It will have to be demonstrated, whether SL dissociation can be corrected in the long-term follow-up using this method. The biomechanical results are very promising.
